# Assessment of Basic Life Support Knowledge Among Medical Students in Jordan: Implications for Improving Out-of-Hospital Cardiac Arrest and Road Traffic Accident Survival Rates

**DOI:** 10.7759/cureus.50080

**Published:** 2023-12-06

**Authors:** Lou'i Al-Husinat, Mokeem Nusir, Haitham Al-Gharaibeh, Mohammad Nusir, Fadi Haddad, Zaid Al Modanat, Giustino Varrassi

**Affiliations:** 1 Department of Clinical Medical Sciences, Faculty of Medicine, Yarmouk University, Irbid, JOR; 2 Department of Clinical Sciences, Faculty of Medicine, Yarmouk University, Irbid, JOR; 3 Clinical Research, Center for Advanced Kidney Research, St Clair Shores, USA; 4 Pain Medicine, Paolo Procacci Foundation, Rome, ITA

**Keywords:** medical education, trauma, knowledge, medical students, basic life support (bls)

## Abstract

This study aimed to assess the level of basic life support (BLS) knowledge among fifth- and sixth-year medical students in Jordan, identify differences in knowledge levels between male and female students and between different universities, and provide insights into the current status of BLS training in medical education in Jordan. The study had 570 respondents, with females constituting 61.1% of the sample. The total average score for medical students was 12.24/17 (72%), and there was a considerable variation in the response rate between universities. The study found that students whose source of knowledge was previous college courses had the highest mean score, and only 24.9% knew the proper position of both hands while doing chest compressions. The study underscores the importance of adequate BLS training for healthcare providers to improve survival rates and reduce mortality and morbidity associated with out-of-hospital cardiac arrest and road traffic accidents. The findings of this study could inform future interventions aimed at improving BLS knowledge and skills.

## Introduction

Cardiac arrest is a significant global public health problem and a leading cause of death worldwide [1]. Out-of-hospital cardiac arrest (OHCA) and road traffic accidents (RTA) are significant contributors to morbidity and mortality globally [2-4]. In Jordan, the survival rate of OHCA is estimated to be 2.97% [5].

Despite the availability of advanced medical interventions, survival rates for OHCA remain low, with less than 10% of all patients surviving [6]. One of the critical factors that impact OHCA survival rates is the time it takes to initiate cardiopulmonary resuscitation (CPR) [7-9]. Early recognition of cardiac arrest and prompt initiation of CPR can significantly improve survival rates and reduce mortality and morbidity associated with OHCA [10].

Similarly, RTA are a major contributor to the global burden of diseases, with an estimated 1.35 million deaths worldwide each year [11]. In 2020, RTA was declared the third-largest contributor to the global burden of diseases [12]. Prompt initiation of CPR by bystanders can significantly reduce mortality and morbidity associated with RTA [13]. Adequate knowledge and skills in basic life support (BLS) are crucial in responding to cardiac arrest and RTA emergencies. Unfortunately, studies have shown that both the general population and healthcare providers lack adequate knowledge and skills in BLS [14,15]. In the Middle East, the lack of adequate BLS training is a significant concern, with a study involving nine Arab countries indicating below desired knowledge [16]. Additionally, a study evaluating public awareness, knowledge, and attitudes towards BLS in Jordan found that only 29% had previous CPR training [17]. While there are several studies addressing the knowledge of BLS among medical students in Jordan, our study distinguishes itself through a unique focus on a specific and critical subset of this population (medical students in their last two years of education), offering an in-depth exploration of a crucial phase in their training.

In this study, we aimed to assess the level of BLS knowledge among fifth- and sixth-year medical students in Jordan. We also aimed to identify any differences in BLS knowledge levels between male and female students and between different universities. The findings of this study could provide valuable insights into the current status of BLS training in medical education in Jordan and could help to inform future interventions aimed at improving BLS knowledge and skills among healthcare providers.

## Materials and methods

To achieve the objectives of this study, we employed a cross-sectional design that allowed us to collect data at a single point in time from a population of fifth- and sixth-year medical students in Jordan. Based on the Krejcie and Morgan sample size calculation formula for a finite population, the minimum recommended sample size was 350 students (using 5% as margin of error and 95% confidence interval). Using a convenience sampling method, we recruited 570 participants from six universities: Yarmouk University, Al-Balqa Applied University, Jordan University of Science and Technology, Hashemite University, Mu’tah University and the University of Jordan. 

Inclusion criteria were medical students in their fifth or sixth year of medical education who were enrolled in medicine at one of the six Jordanian universities. Exclusion criteria encompassed students from non-medical majors and those within the first four years of medical education. 

Data were collected using an online self-administered questionnaire. In pursuit of validity, we conducted content validity testing via multiple faculty members specialized in the matter of interest. We also used pilot-testing during which we distributed the questionnaire to multiple students prior to the official process of data collection. Test-retest reliability was also assessed by redistributing the questionnaire to a subset of the sample consisting of 40 fifth- and sixth-year medical students with a four-week interval between administrations. The intraclass reliability coefficient (ICC) obtained was 0.85, indicating good reliability. Our questionnaire consisted of three parts. The first part included demographic information such as study year, gender, and university. The second part contained questions related to BLS knowledge, including the source of knowledge, the proper position of hands during chest compressions, the first sign to look for during airway assessment of an unconscious patient, etc. The third part included an optional open-ended question in order to explore suggestions about BLS knowledge improvement. Table 1 displays the full questionnaire.

**Table 1:** Test questions for assessing students' knowledge This questionnaire was developed by the authors of this study.

Gender:
Medical studying year:
University:
What does BLS stand for? (1 point) Basic Life Support Basic Life Safety Basic Life Survival
If your answer was A, please mention your primary source of knowledge of BLS. (0 points) University courses that you have taken YouTube Social media Television Movies Relatives/friends
What is the lowest possible value of Glasgow Coma Scale? (1 point) 0 3 1 2
What constitutes the summative score of the Glasgow Coma Scale? (1 point) 13 14 15 10
What is the primary action that a bystander or witness should take when encountering an unresponsive individual who exhibits no signs of normal respiration? (1 point) Check his/her vitals Call 911 Start chest compressions Tilt the victim to the left side
What does the ABCDE mnemonic stand for? (1 point)
What does the letter C stand for? (1 point)
What is the first sign to look for during airway assessment? (1 point)
What is the precise anatomical location for performing chest compressions with regard to cardiopulmonary resuscitation (CPR)? (1 point) Right hemithorax Center of the chest just below the nipples Xiphisternum At the apex of the heart corresponding to the maximum apical impulse
What is the proper rate of chest compressions? (1 point) 1/second 2/second 30/minute 45/minute
What is the proper depth of chest compressions? (1 point) 1 cm 2 cm 2 Inches (5cm) 3 Inches (7cm)
What is the recommended technique for hand placement during the execution of chest compressions (in adults)? (specifically pertaining to the positioning of the dominant and non-dominant hands) (1 point) Placing your dominant hand above your non-dominant hand, while kneeling at the right side of the victim. Placing your non-dominant hand above your dominant hand, while kneeling at the right side of the victim. Placing both hands in a parallel fashion. Placing your non-dominant hand above your dominant hand, while kneeling near the victim’s head.
Which of the following complications are frequently encountered during chest compressions? (1 point) Rib fractures and sternal injuries Traumatic pneumothorax Shoulder dislocation Cardiac tamponade
What is the initial step to undertake when presented with a penetrating trauma injury, and the foreign object remains embedded within the wound? (1 point) Do not attempt to remove the object and stop any visible external bleeding Remove the object and compress the area of bleeding until it stops Elevate legs above body level Put direct pressure on the object to stop bleeding
Which maneuver should be used when suspecting upper airway obstruction? (1 point) Head tilt-chin lift Jaw-thrust Rotating victim’s head to the left Elevating victim’s head above body level
Which of the following is considered a sign of Tension Pneumothorax? (1 point) Distended neck veins Collapsed neck veins Dull percussion on the affected side Crepitus on palpation
Next step in management when suspecting tension pneumothorax? (1 point) Immediate needle decompression followed by chest tube insertion Immediate needle decompression only Intubation and mechanical ventilation Establish IV line to prepare for fluid resuscitation and possible drug administration
What is the recommended course of action to take when observing a patient exhibiting tonic-clonic convulsions? (1 point) Providing safety environment and tilting the victim on his/her side Attempting to stop the victim’s movements Obtaining his/her vital signs and oxygen saturation Providing safety environment and try to put the victim in a prone position Watch and wait
What strategies or recommendations can be proposed to enhance the Basic Life Support (BLS) knowledge of medical students ………………………………………………………………………………………………………………………………………….
Total score (__/17)

Prior to data collection, ethical approval was obtained from the institutional review board at Yarmouk University (DSR/2023/469). Participation in the study was voluntary, and participants provided informed consent before completing the questionnaire. To ensure confidentiality, participants were assigned a unique identification number, and data were collected anonymously.

Data were analyzed using SPSS version 25.0 (IBM Corp., Armonk, NY, USA). Descriptive statistics were employed to summarize demographic data and BLS knowledge scores. ANOVA tests were utilized to compare mean scores of demographic variables with multiple categories (e.g., universities, knowledge sources, etc.). Two-sample t-tests were applied to compare mean scores of binary categorical variables, such as gender (male or female) and study year (fifth or sixth). The decision to use ANOVA and t-tests was based on the distribution of variables and the nature of the comparisons being made. A p-value of less than 0.05 was considered statistically significant.

## Results

The total estimated number of fifth- and sixth-year medical students approached was 3922. However, exact population size was challenging due to the dynamic nature of student enrollment. A total of 570 medical students participated in this study (response rate 14.5%), with females accounting for 61.1% (348) of the sample and males comprising 38.9% (222), resulting in a female-to-male gender ratio of 1.56:1.00. No significant difference was observed in the number of respondents between fifth- and sixth-year medical students. Yarmouk University had the highest number of respondents in the study sample (175, 30.7%), followed by Al-Balqa Applied University (107, 18.8%). A complete breakdown of the demographic details can be found in Table 2. However, there was a substantial variation in the response rate between universities, with Yarmouk University having the highest response rate of 43%, while the University of Jordan had the lowest response rate of 7.6%.

**Table 2:** Demographic characteristics of study participants

Parameters	Sample Size	Proportion of Sample
University		
Yarmouk University	175	30.7%
Al-Balqa Applied University	107	18.8%
Hashemite University	81	14.2%
Mu’tah University	75	13.2%
Jordan University of Science and Technology	71	12.5%
University of Jordan	61	10.7%
Medical Year		
5^th^	296	51.9%
6^th^	274	48.1%
Gender		
Male	222	38.9%
Female	348	61.1%

Overall, the mean score for medical students' BLS knowledge was 12.24/17 (72%), with a standard deviation of 2.113. The range of BLS knowledge scores varied widely among students from different universities, with a significant difference observed between the means and standard deviations (F (5,564) = 5.21, p-value 0.018). The University of Jordan students had a mean score of 12.70, while Al-Balqa Applied University students had a mean of 11.70. Table 3 provides a detailed comparison between universities regarding BLS knowledge.

**Table 3:** Basic life support (BLS) knowledge scores among medical students in Jordan P-value <0.05 suggests statistically significant differences in mean knowledge scores between universities. Total range of BLS knowledge score is 4-16

University	Sample Size	Mean scores	Standard Deviation of Scores	P-value
Yarmouk University	175	12.25	2.021	0.018
Al-Balqa Applied University	107	11.70	2.496	
Hashemite University	81	12.09	1.879	
Mu’tah University	75	12.51	1.766	
Jordan University of science and technology	71	12.56	1.547	
University of Jordan	61	12.70	2.679	

There was also a considerable difference between universities regarding the source of students' BLS knowledge, with only 46.9% of Yarmouk University students indicating that their source was previous college courses, whereas students from other universities, such as Hashemite University, reported 80.2%. Table 4 displays a comparison between universities regarding the source of BLS knowledge.

**Table 4:** University courses as the sole source of basic life support (BLS) knowledge

University	Proportion of respondents who acquired BLS knowledge from college courses
Yarmouk University	82 (46.9%)
Al-Balqa Applied University	68 (63.6%)
Hashemite University	65 (80.2%)
Mu’tah University	42 (56%)
Jordan University of science and technology	46 (64.8%)
University of Jordan	47 (77%)

No significant association was observed between gender and BLS knowledge (t (568) = 1.25, p-value >0.05), nor was there a significant association between the year of study (fifth and sixth) and BLS knowledge (t (568) = 0.75, p-value >0.05). However, a significant difference was noted in the scores of students who obtained their BLS knowledge from different sources (F (4, 565) = 6.78, p-value =0.001). Students who obtained their knowledge from previous college courses had the highest mean score (12.74) compared to those who relied on other sources such as YouTube, social media, friends/relatives, and movies. Table 5 shows the mean and standard deviation of students for each knowledge source.

**Table 5:** Comparison of mean scores among five different sources of knowledge

Source of Knowledge	Mean scores	Standard Deviation	P-value
University courses	12.74	1.818	0.001
YouTube	11.88	2.311	
Social Media	12.00	1.905	
Relatives/Friends	12.44	2.404	
Movies	11.50	3.162	

**Figure 1:**
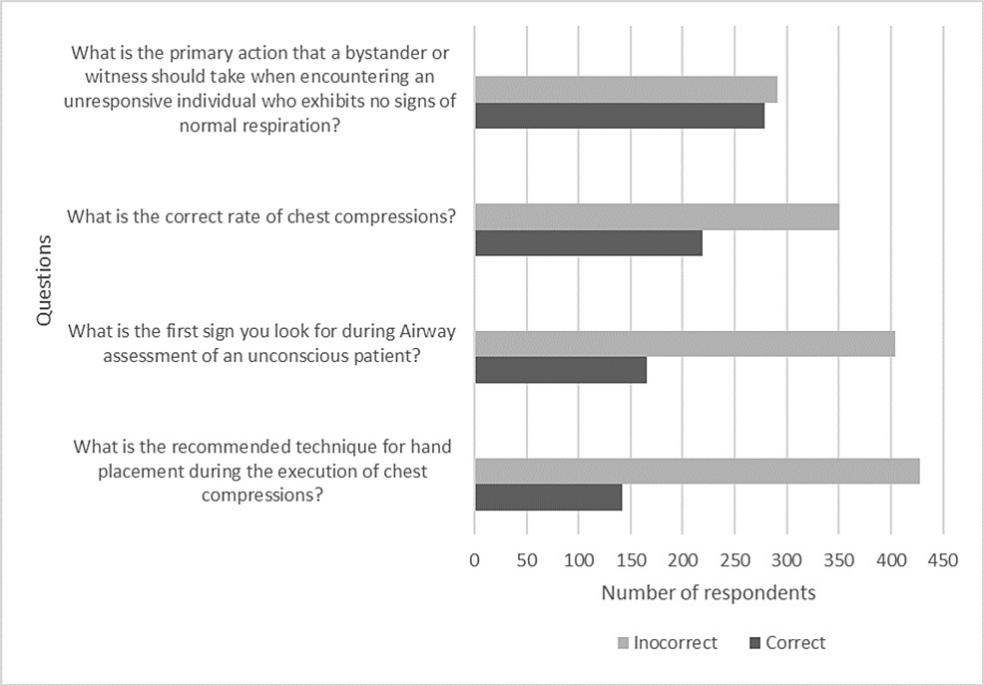
Questions with the most incorrect answer percentage

Unfortunately, the findings reveal that only 24.9% of participants knew the proper hand position for chest compressions, 29.1% knew the first sign to look for during airway assessment of an unconscious patient, and 38.4% knew the proper rate of chest compressions. Figure 1 lists frequently missed questions.

## Discussion

According to recent American Heart Association (AHA) guidelines, the least expected score in the BLS subject is 84% [18]. Despite this, our study showed that a considerable number of medical students in Jordan had insufficient knowledge of BLS. While other studies have been conducted about BLS knowledge in Jordan, our study is unique in its focus on medical students, who are expected to have greater knowledge of BLS. Unfortunately, studies indicate that Middle Eastern countries, including Jordan, lack sufficient training for both medical students and the general population. For example, a study conducted among female health students at a women's university in Saudi Arabia showed that the mean overall knowledge score was very low, with 87.9% of participants having low knowledge scores [15]. Similarly, our survey results among fifth- and sixth-year medical students in Jordan showed lower-than-expected scores.

Gender was not found to be a significant factor in BLS knowledge among medical students in Jordan. While some studies have found significant gender differences [19,20], our results were consistent with other studies showing no such association [21]. However, willingness to perform CPR did vary significantly by gender, with females reporting less willingness to perform resuscitation [22].

We found significant variation in BLS knowledge between different universities, likely due to differences in the curriculum. Studies have shown that additional BLS training modules can significantly improve BLS knowledge in medical students [23-27], highlighting the importance of such training in medical education. Our study identified several areas of weakness in BLS knowledge among medical students in Jordan, including proper chest compression rate in adults. These weaknesses are not unique to Jordanian medical students and are a common issue worldwide [28].

Our study has some limitations that should be considered. First, our assessment of BLS knowledge was based solely on multiple-choice questions, and therefore, we cannot draw conclusions about practical BLS knowledge. Second, our response rate varied widely between universities, with some universities having very low response rates. Finally, the limited number of studies on this topic in Jordan makes it challenging to draw comparisons and reach definitive conclusions.

## Conclusions

The current study provides important data regarding the level of knowledge concerning BLS among fifth- and sixth-year medical students in Jordanian medical faculties. Our findings indicate that students from different universities in Jordan have different levels of BLS knowledge. Moreover, the study revealed a lower-than-anticipated score trend. Establishing comprehensive and mandatory BLS courses in all universities might help ensuring that medical students are well-trained in BLS principles.

## References

[1] Mozaffarian D, Benjamin EJ, Go AS (2016). Heart disease and stroke statistics-2016 update: a report from the American Heart Association. Circulation.

[2] (2023). Global Health Estimates: Life expectancy and leading causes of death and disability. https://www.who.int/data/gho/data/themes/mortality-and-global-health-estimates.

[3] (2023). Global status report on road safety. https://www.who.int/publications-detail-redirect/9789241565684.

[4] Khurshid A, Sohail A, Khurshid M, Shah MU, Jaffry AA (2021). Analysis of road traffic accident fatalities in Karachi, Pakistan: an autopsy-based study. Cureus.

[5] Raffee LA, Samrah SM, Al Yousef HN, Abeeleh MA, Alawneh KZ (2017). Incidence, characteristics, and survival trend of cardiopulmonary resuscitation following in-hospital compared to out-of-hospital cardiac arrest in northern Jordan. Indian J Crit Care Med.

[6] Gräsner JT, Lefering R, Koster RW (2016). EuReCa ONE-27 Nations, ONE Europe, ONE Registry: a prospective one month analysis of out-of-hospital cardiac arrest outcomes in 27 countries in Europe. Resuscitation.

[7] Ong ME, Perkins GD, Cariou A (2018). Out-of-hospital cardiac arrest: prehospital management. Lancet.

[8] Gopalakrishnan S (2012). A public health perspective of road traffic accidents. J Family Med Prim Care.

[9] Sasson C, Rogers MA, Dahl J, Kellermann AL (2010). Predictors of survival from out-of-hospital cardiac arrest: a systematic review and meta-analysis. Circ Cardiovasc Qual Outcomes.

[10] Atwood C, Eisenberg MS, Herlitz J, Rea TD (2005). Incidence of EMS-treated out-of-hospital cardiac arrest in Europe. Resuscitation.

[11] (2023). Global status report on road safety 2018. https://www.who.int/publications/i/item/9789241565684.

[12] Murray CJ (2022). The global burden of disease study at 30 years. Nat Med.

[13] Ibrahim WH (2007). Recent advances and controversies in adult cardiopulmonary resuscitation. Postgrad Med J.

[14] Baldi E (2021). Lack of cardiopulmonary resuscitation knowledge among young medical doctors: a worldwide issue. Indian J Crit Care Med.

[15] Al-Mohaissen MA (2017). Knowledge and attitudes towards basic life support among health students at a Saudi women's university. Sultan Qaboos Univ Med J.

[16] Shaheen N, Shaheen A, Diab RA (2023). Basic life support (BLS) knowledge among general population; a multinational study in nine Arab countries. Arch Acad Emerg Med.

[17] Jarrah S, Judeh M, AbuRuz ME (2018). Evaluation of public awareness, knowledge and attitudes towards basic life support: a cross-sectional study. BMC Emerg Med.

[18] (2023). BLS Online Exam for Instructor-led Training. https://elearning.heart.org/course/416.

[19] Alkandari SA, Alyahya L, Abdulwahab M (2017). Cardiopulmonary resuscitation knowledge and attitude among general dentists in Kuwait. World J Emerg Med.

[20] Mekonnen CK, Muhye AB (2020). Basic life support knowledge and its associated factors among a non-medical population in Gondar Town, Ethiopia. Open Access Emerg Med.

[21] Abbas F, Sawaf B, Hanafi I (2018). Peers versus professional training of basic life support in Syria: a randomized controlled trial. BMC Med Educ.

[22] Krammel M, Schnaubelt S, Weidenauer D (2018). Gender and age-specific aspects of awareness and knowledge in basic life support. PLoS One.

[23] Saquib SA, Al-Harthi HM, Khoshhal AA (2019). Knowledge and attitude about basic life support and emergency medical services amongst healthcare interns in university hospitals: a cross-sectional study. Emerg Med Int.

[24] Lešnik D, Lešnik B, Golub J, Križmarić M, Mally S, Grmec S (2011). Impact of additional module training on the level of basic life support knowledge of first year students at the University of Maribor. Int J Emerg Med.

[25] Srivilaithon W, Amnuaypattanapon K, Limjindaporn C, Diskumpon N, Dasanadeba I, Daorattanachai K (2020). Retention of basic-life-support knowledge and skills in second-year medical students. Open Access Emerg Med.

[26] Lami M, Nair P, Gadhvi K (2016). Improving basic life support training for medical students. Adv Med Educ Pract.

[27] Lee JH, Cho Y, Kang KH, Cho GC, Song KJ, Lee CH (2016). The effect of the duration of basic life support training on the learners' cardiopulmonary and automated external defibrillator skills. Biomed Res Int.

[28] Mohammed Z, Arafa A, Saleh Y (2020). Knowledge of and attitudes towards cardiopulmonary resuscitation among junior doctors and medical students in Upper Egypt: cross-sectional study. Int J Emerg Med.

